# Diaper dermatitis: a survey of risk factors in Thai children aged under 24 months

**DOI:** 10.1186/s12895-019-0089-1

**Published:** 2019-07-02

**Authors:** Chonnakarn Sukhneewat, Jitjira Chaiyarit, Leelawadee Techasatian

**Affiliations:** 1 Pediatric Department, Faculty of Medicine, Khon Kaen University, Khon Kaen, Thailand; 2 Clinical epidemiology unit, Faculty of Medicine, Khon Kaen University, Khon Kaen, Thailand; 3 Dermatology Division, Pediatric Department, Faculty of Medicine, Khon Kaen University, Khon Kaen, 40002 Thailand

**Keywords:** Baby talcum powder, Dermatitis, Diaper dermatitis, Risk factor

## Abstract

**Background:**

To identify the prevalence and risk factors associated with diaper dermatitis in Thai children aged 1–24 months.

**Methods:**

This was a cross-sectional study of 1153 participants using structural questionnaires, which was conducted at Khon Kaen University Faculty of Medicine Pediatric department in Thailand. Univariate and multivariate logistic regression analyses were used to test the association between diaper dermatitis and its possible risk factors.

**Results:**

The prevalence of diaper dermatitis among the study population was 36.1%, a rate which significantly decreased with age. The highest prevalence was found in subjects who were one to six months old. Risk factors that had a statistically significant association with diaper dermatitis in both univariate and multivariate analysis were i) diaper changing fewer than three times/night, ii) previous episodes of diaper rash, iii) using cloth diapers, and iv) topical application of baby talcum powder to the diaper area.

**Conclusions:**

Frequent diaper changings during the daytime do not compensate for fewer changings during the night. Moreover, the use of baby talcum powder on the diaper area significantly increased the risk of diaper dermatitis among the study population. These findings should be applied in future preventive strategies for diaper dermatitis in this age group.

**Electronic supplementary material:**

The online version of this article (10.1186/s12895-019-0089-1) contains supplementary material, which is available to authorized users.

## Background

Diaper dermatitis refers to any clinical sign of skin inflammation that occurs on the area covered by a diaper. Most skin inflammation is irritation caused by moisture, heat, or various enzymes from urine and feces, which are major irritants to the skin [1–4]. There is a high prevalence of diaper dermatitis among children who require diapering. The estimated overall prevalence has been found to range from 7 to 43.8% and varies according to setting, hygiene practices, and age group [5]. Children under 24 months old have the highest prevalence, with the peak being between nine and 12 months of age. This is likely due to the fact that the children in this age group require diapering [3] at a greater rate than those in other age groups. There are many risk factors that have been found to increase the occurrence of diaper dermatitis. These factors included gastrointestinal tract infection, the type of diaper used, and the frequency of diaper changes [6]. There is little information available on diaper dermatitis in Thailand. This study was, thus, developed to explore the prevalence and risk factors of diaper dermatitis among Thai children in this age group (1–24 months). The results from this present study will have application in the future development of preventive strategies for diaper dermatitis in Thai children.

## Methods

This cross-sectional study was conducted at the Khon Kaen University, Faculty of Medicine, Pediatric Department, in the Well Baby Clinics between November 2015 and January 2017. All children aged 1–24 months were eligible.

Consecutive cases entered into the Well Baby Clinics were asked to participate in the study. A total number of 1153 children were enrolled based on our sample size calculation. The diagnosis of diaper dermatitis was made based on parental reporting of any skin rashes on the diaper area during the past 6 weeks. The demographic background information included in the structural questionnaires was age, sex, general condition, and underlying atopic diseases. The possible risk factors of diaper dermatitis, including gastrointestinal tract infection, type of diaper used, and frequency of diaper changes, were also addressed in a structural questionnaire. A detailed description of the questionnaire is included in an Additional file 1.

At the end of the study, the collected data were analyzed using STATA software version 10 (StataCorp LP). Descriptive statistical methods – means, standard deviations (SDs), medians and frequencies – were used to analyze the demographic data. Univariate and multivariate logistic regression analyses were performed to test the associations between the proposed factors and diaper dermatitis. The authors first estimated the association between each risk factor and diaper dermatitis (bivariate analysis). The initial multivariate analysis model included all risk factors. Independent risk factors for diaper dermatitis were identified by forward stepwise logistic regression where all univariate predictors with a *p*-value < 0.05 were included. Multivariate regression was corrected for multiple testing. Values of *P* < 0.05 were considered to indicate statistical significance. Incomplete questionnaires and missing data were addressed as imputed data, and final calculations included all recorded data.

The study was approved by the institutional review board of the Khon Kaen University, Human Ethical Committee (#HE581286). Before participants were enrolled in the study, written informed consent was obtained from their parents or guardians. The study was funded by a grant from the Khon Kaen University, Faculty of Medicine in Thailand: (Grant Number IN58332).

## Results

**Fig. 1:**
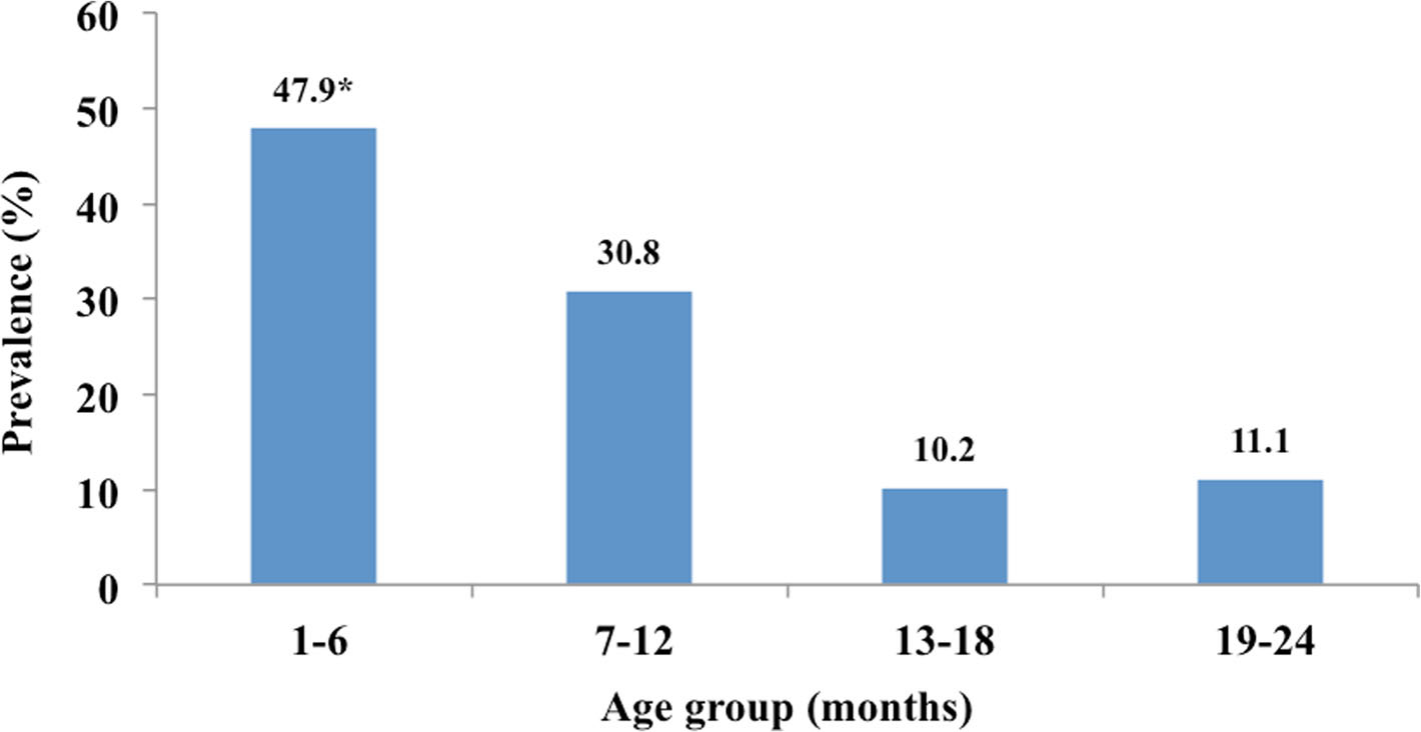
Variation in the prevalence of diaper dermatitis among different age groups

A total of 1153 children were recruited for the study: 585 (50.7%) boys and 568 (49.3%) girls. Their ages ranged from 1 to 24 months (median = six months) with a mean age of 8.7 months (SD 4.3). The prevalence of diaper dermatitis was 36.1% (416/1153). The highest prevalence was among the children who were 1–6 months old (47.9%). This age group also had a significantly higher prevalence of diaper dermatitis than the other three age groups (7–12, 13–18 and 19–24 months; *P* < 0.05). Figure 1 represents the variation in the prevalence of diaper dermatitis among the different age groups. There were no significant differences in term of prevalence between sexes (boys 37.4% [219/585] versus girls 34.5% [249/721]; *P*-value = 0.31).

Univariate analysis showed that diaper dermatitis was significantly associated with the following five factors: i) underlying allergic diseases, ii) fewer than three diaper changes per night, iii) previous episodes of diaper rash, iv) use of cloth diapers, and v) use of baby talcum powder on the diaper area when diaper dermatitis is present (Table 1). Four of the five factors that were significantly associated with diaper dermatitis according to univariate logistic regression analysis were also significantly related according to multivariate logistic regression analysis. (factors ii trough v; *P* < 0.001 for all comparisons; Table 2).

**Table 1:** Summary of the factors associated with diaper dermatitis in the study population

Factors	Diaper dermatitis		Univariate logistic regression		
	Yes *n* = 416	No *n* = 737	OR	95% CI	*P*-value
Age					< 0.001*
1–6 months	239 (47.99)	259 (52.01)	1	0.37, 0.64	
7–12 months	122 (30.81)	274 (69.19)	0.48	0.18, 0.41	
13–18 months	35 (20.23)	138 (79.77)	0.28	0.19, 0.56	
19–24 months	20 (23.26)	66 (76.74)	0.33	0.37, 0.64	
Children with underlying allergic diseases					< 0.001*
No	317 (33.54)	628 (66.46)	1		
Yes	99 (47.60)	109 (52.40)	1.8	1.33, 2.44	
Type of diaper using					< 0.001*
Disposable diaper [40.4% (466/1153)]	79 (16.95)	387 (83.05)	1		
Cloth diaper [59.6% (687/1153)]	337 (49.05)	350 (50.95)	4.72	3.55, 6.27	
Previous episodes of diaper dermatitis					< 0.001*
None	19 (6.96)	254 (93.04)	1		
≤ 2 times	258 (51.09)	247 (48.91)	13.96	8.49, 22.97	
> 2 times	139 (37.07)	236 (62.930	7.87	4.72, 13.13	
Frequency of diaper changes					
Total number/day					0.717
≥ 6 times	159 (35.49)	289 (64.51)	1		
< 6 times	254 (36.55)	441 (63.45)	1.05	(0.82, 1.34)	
Total number/daytime					0.765
≥ 3 times	155 (35.63)	280 (64.37)	1		
< 3 times	261 (36.50)	454 (63.50)	1.04	0.81, 1.33	
Total number/nighttime					< 0.001*
≥ 3 times	16 (12.21)	115 (87.79)	1		
< 3 times	387 (38.82)	610 (61.18)	4.56	(2.66, 7.81)	
Topical application on diaper area					< 0.001*
Diaper cream	46 (29.30)	111 (70.70)	1		
Baby powder	173 (40.42)	255 (59.58)	1.64	1.10, 2.43	
Baby lotion	9 (17.31)	43 (82.69)	0.51	0.23, 1.12

**Table 2:** Summary of the five factors that were found to be significantly associated with diaper dermatitis in Thai children aged 1–24 months according to multivariate logistic regression analysis

Factors	Univariate logistic regression		Multivariate logistic regression		
	OR	95% CI	OR	95% CI	*P*-value
Age					
1–6 months	1		1		
7–12 months	0.48	0.37, 0.64	0.35	0.25, 0.49	< 0.001*
13–18 months	0.28	0.18, 0.41	0.15	0.09, 0.24	< 0.001*
19–24 months	0.33	0.19, 0.56	0.31	0.17, 0.58	0.001*
Type of diaper using					
Disposable diaper	1		1		
Cloth diaper	4.72	3.55, 6.27	5.47	3.78, 7.92	< 0.001*
Previous episodes of diaper dermatitis					
None	1		1		
≤ 2 times	13.96	8.49, 22.97	12.16	6.72, 22.01	< 0.001*
> 2 times	7.87	4.72, 13.13	9.71	5.28, 17.86	< 0.001*
Topical application on diaper area					
Diaper cream	1		1		
Baby powder	1.64	1.10, 2.43	1.61	1.04, 2.57	0.005*
Baby lotion	0.51	0.23, 1.12	2.59	1.37, 9.39	0.009*
Total number of diaper changing/nighttime					
≥ 3 times	1		1		
< 3 times	4.56	(2.66, 7.81)	3.72	2.04, 6.76	< 0.001*

Underlying allergic diseases (atopic dermatitis, allergic rhinitis, asthma and cow milk protein allergies) were documented as having significant association with diaper dermatitis according to univariate logistic regression analysis. However, further multivariate logistic regression calculation could not be performed for each individual allergic condition because the sample size in each of these groups was too small. Other factors that were listed in the structural questionnaires but had no association with increased prevalence of diaper dermatitis were parental age, parental education, child’s weight, and type of food consumed during the past 6 weeks.

## Discussion

To our knowledge, this study is the first report on the prevalence of diaper dermatitis in Thai children. The age of the study population ranged from 1 to 24 months, as previous studies have found children in this age range to have a high prevalence of diaper dermatitis [5, 7–9]. The high prevalence is due to the fact that children in this age range still need diapering. Most children over two years of age are able to use the toilet by themselves, decreasing the need for diapering substantially.

The prevalence of diaper dermatitis in the present study was 36.1%. This number was similar to those found in previous studies, which have ranged from 7 to 43.8%. There was no difference in the prevalence of diaper dermatitis between boys and girls, a finding that is similar to those of other studies in Asian [10], North American, and European countries [5, 9, 11].

The present study classified the children into four age groups: 1–6, 7–12, 13–18 and 19–24 months. The highest prevalence was found among the children aged 1–6 months with a significantly higher prevalence than the other three age groups (*P* < 0.001).

The standard recommendation is that diapers should be changed every three to four hours, a duration that is based on the frequency of urination in infants [1–3, 12]. This means that diapers should be changed from six to eight times/day. This is one of the major factors that influence the prevalence of diaper dermatitis, as the condition becomes more likely when there is prolonged contact of urine and feces with the skin. Previous studies have shown that the prevalence of diaper dermatitis is significantly higher when diapers are changed fewer than six times/day compared with more frequent changing [5]. The present study went further and compared the effects of daytime changings versus nighttime changings on the prevalence of diaper dermatitis. We found that fewer than three changes/night led to a significant increase in the risk of diaper dermatitis compared to more frequent changings (multivariate logistic regression analysis, OR = 3.72, *P* < 0.001). However, fewer than three changings during the daytime did not lead to significant increases in diaper dermatitis, meaning that more frequent daytime changings cannot compensate for fewer changings during the night. These findings support the idea that it is important to change an infant’s diaper after each time he/she urinates.

In the present study, 40.4% (466/1153) of participants used only disposable diapers, and 59.6% (687/1153) used only cloth diapers. The authors found that the rate of diaper dermatitis was significantly higher in the participants who used cloth diapers compared to those who used disposable diapers, (multivariate analysis OR = 5.47, *P* < 0.001). This is consistent with the results of previous studies conducted after absorbent gel technology had become widely used in disposable diapers [9, 13–15].

Although a Cochrane review [16] could not find enough evidence from good-quality randomized controlled trials to support or refute the benefit of disposable diapers in the prevention of diaper dermatitis in infants, their use is recommended for all infants. This is supported by evidence that a new absorbent gel technology in disposable diapers is effective in drawing urine away from the diaper area and keeping the skin dry, reducing dermatological problems in the diaper area [17–19].

All of the disposable diaper brands used by participants in this study (MamyPoko, BabyLove, Goon, and Huggies), used the newly developed absorbent gel technology mentioned above. This may explain the significantly lower rate of diaper dermatitis in participants who used disposable diapers compared to those who used cloth diapers.

It is well known that using diaper creams that enhance the skin barrier is an effective strategy for preventing diaper dermatitis [20–22]. The major properties of over-the-counter diaper creams that help prevent diaper rash are as follows: i) containing fragrance-free moisturizer to restore skin barrier function, ii) exhibiting a barrier effect to protect the skin from major irritants (urine, feces), and iii) containing dexpanthenol (B5), which has been proven to be effective in treating skin inflammation on the diaper area [21, 22]. In Thailand, there are many effective diaper creams that are readily available over the counter. However, baby talcum powder is more frequently used for this purpose. The present study showed that, 37.1% (428/1153) of the study population used baby powder on the diaper area, with only 13.6% (157/1153) using an effective diaper cream.

Topical application of baby talcum powder is thought to be useful in keeping the skin dry [11, 23, 24]. However, it has only been found to be beneficial in cases of uncomplicated diaper dermatitis and not in more severe cases of cutaneous inflammation [25]. In addition, the application of talcum powder to areas of the skin where the epidermal barrier is absent or significantly disrupted or to moist areas can cause skin irritation [13, 15, 26]. The most serious possible side effect of baby talcum powder that has been reported is respiratory distress from accidental massive aspiration [27]. Moreover, another report found that prolonged inhalation of talcum powder can induce surfactant depletion, bronchiolar hyper-responsiveness, and alveolar damage [28]. According to these reports, baby talcum powder should be used only to preserve skin dryness to a limited extent on certain areas. The present study also found that the use of baby talcum powder significantly increased diaper dermatitis when compared to diaper creams in the study population (multivariate logistic regression analysis, OR = 1.61, *P* = 0.005). Thus, the use of diaper creams rather than baby talcum powders should be encouraged in the Thai population.

There were several potential limitations to the present study. The questionnaire was only structured to examine possible risk factors for diaper dermatitis. However, there may be other unknown factors that have an impact on diaper dermatitis that were not included in the analyses. In addition, some of the respondents did not complete the sections in the questionnaires regarding underlying atopic diseases and economic status. The authors addressed the missing information as imputed data and calculated the recorded data all together. This may have affected the power of the test, but the main possible risk factors were record completely.

## Conclusions

Changing diapers fewer than three times/night and using baby talcum powder increased the risk of diaper dermatitis among the study population. Therefore, future strategies to prevent diaper dermatitis in this age group should emphasize frequent diaper changing (especially at night) and the application of diaper cream containing dexpanthenol (B5) instead of baby talcum powder on the diaper area.

## Additional file

Additional file 1: A structural questionnaire. A detailed description of the questionnaire. (DOCX 66 kb)

## Data Availability

The datasets used and/or analysed during the current study are available from the corresponding author on reasonable request.
