# Supplementary material for: Diaper dermatitis: a survey of risk factors in Thai children aged under 24 months
Source: BMC Dermatol. 2019 Jul 2;19:7. doi: 10.1186/s12895-019-0089-1 (PMC6604136; doi:10.1186/s12895-019-0089-1)
Supplement: Supplementary file 1 — A structural questionnaire. A detailed description of the questionnaire. (DOCX 66 kb) [file 12895_2019_89_MOESM1_ESM.docx]

**Detailed outline of the questionnaire**

(Original version is in Thai language)

**Diaper dermatitis: a survey of risk factors in Thai children aged under 24 months**

**Demographic characteristics**

- Age
- Sex
- Current body weight
- Underlying Atopic disease

*If YES*

- - - - Skin
      - Respiratory
      - Gastrointestinal tract
- Primary care giver
  - - - Parents
      - Others
- Age of the primary care giver
- Educational level of the primary care giver
- Career of the primary care giver

**Possible associating factors**

- Experience diaper rash during the past 6 weeks?
- Previous experience of diaper rash?

*If YES …* How often?

- Diaper rash at the time having gastrointestinal tract infection?
- How do you treat diaper rash?
- None
  - - - Barrier cream
      - Baby powder
      - Topical medication (specify…)
      - Doctor prescription
- How does the stool look like?
  - - - Normal/ soft
      - Loose
      - Hard
      - Watery
      - Vary between loose and watery
- How often does your child stool?
  - - - 1-2 time/day
      - > 3 times/day
      - < 3 times/week
- How often do you change the diaper during daytime?
  - - - < 3 times
      - > 3times
- How often do you change the diaper during nighttime?
  - - - < 3 times
      - > 3times
- What type of food during the past 6 weeks?
  - - - Breast milk
      - Cow milk (Specify)
      - Breast milk and cow milk (Specify)
      - Banana
      - Soft-boiled rice
      - Others (Specify)

**Types of diaper**

- What type of diaper does your child use?
  - - - None
      - Cloths
      - Disposable
      - Both
- The reason of using cloths diaper
  - - - Safe
      - Cheap
      - Get used to
      - Don’t know
- The reason of using disposable diaper
  - - - Convenience
      - Clean
      - Get used to
      - Don’t know
- What do you use for cleansing on the diaper area of your child?
  - - - Tap water
      - Warm water
      - Boiled water
      - Normal saline
      - Soap, *If YES, specify……*
      - Tissue paper
      - Alcohol
- What are the regular materials that put on the diaper area of your child?
  - - - None
      - Diaper cream
      - Baby powder
      - Regular moisturizer
- What are you put on the diaper area of your child when he/she having diaper rash?
  - - - None
      - Diaper cream
      - Baby powder
      - Regular moisturizer
- When will you bring your child to see the doctor if he/she have diaper rash?
  - - - As early as possible
      - After no improvement within 1-2 days
      - After no improvement within 7 days
      - After no improvement within 14 days
- What is your decision of using disposable diaper?
  - - - Price
      - Quality
      - Design
      - Advertisement
      - Promotion
- What is/are the disposable diaper brands does your child use?

*If YES, specify……*
